# Supplementary material for: Bacterial meta-analysis of chicken cecal microbiota
Source: PeerJ. 2021 Jan 5;9:e10571. doi: 10.7717/peerj.10571 (PMC7792525; doi:10.7717/peerj.10571)
Supplement: Supplemental Information 2 [file peerj-09-10571-s002.docx]

**Meta-analysis rationale**

1. **The rationale for conducting the systematic review / meta-analysis.**

Bacterial composition of the chicken gut has been stablished as a main factor in health and grow of chickens, which has led to the formulation of multiple projects regarding this topic. However, most of those projects are intended to explain the variation of the bacterial composition within the chicken gut, given certain experimental conditions. Experiments describing variations on diet, breed and a broad catalogue of treatments for improving chickens’ conditions have been published, leading to the generation of a high amount of data, which is now available on public databases. Nonetheless, very few studies have tried to put this data together in order to generate a taxonomic consensus of the chicken gut composition. Given the biases related to merging data from multiple sequencing technologies, previous studies have focused on a single technology. In this sense, studies based on Sanger and 454 sequencing technologies are already available, but no meta-analysis have focused on data from Illumina sequencing, which opens a new frontier on this topic. Since Illumina technology has been crucial for the advance on metagenomics in the past decade and is currently the *de facto* method for amplicon-based community analysis, we believe that is important to generate a new study that encompasses all this new information. When comparing this analysis with the previous reports we could be able to reduce the biases regarding taxonomic composition. We expect that this project serves as a comparison tool for new researchers aiming to characterize chicken cecal microbial communities under different treatment conditions.

1. **The contribution that it makes to knowledge in light of previously published related reports, including other meta-analyses and systematic reviews.**

As it was described above, the use of Illumina sequencing data to describe a bacterial consensus of the chicken’s gut has not been performed before. The amount of data generated by this technology surpasses other technologies, and in the case of metagenomic studies, leads to a greater resolution and recovery of bacterial species. Thus, the current study aims at creating a baseline that will have more impact than currently available meta-analysis. Moreover, the characterization methods used in previous meta-analysis are based on an OTU approach by clustering sequences at 97% of identity, a method that in recent years has been criticized due to its tendency to a) create false OTUs due to sequencing errors and b) cluster into a single OTU highly similar sequences but derived from different species . In this study we used an approach based on ASVs, which allows us to differentiate sequences by single nucleotides patterns, increasing the detection rate, coupled with sequencing error correction that reduces false positives in variant identifications. Furthermore, we applied a method, that is not described on previous meta-analysis of this topic. By collapsing taxa at genus level, we were able to diminish the biases inherent to each study. Finally, previous works are focused on making a consensus of the taxonomic composition. Here we preset an analysis that, in addition to the taxonomic characterization, relies on a core microbiome analysis and the application of diversity metrics by clustering data into different categorical variables shared by multiple studies.
